# Preliminary Characterization of Genipin-Cross-Linked Silk Sericin/Poly(vinyl alcohol) Films as Two-Dimensional Wound Dressings for the Healing of Superficial Wounds

**DOI:** 10.1155/2013/904314

**Published:** 2013-09-11

**Authors:** Tippawan Siritientong, Juthamas Ratanavaraporn, Teerapol Srichana, Pornanong Aramwit

**Affiliations:** ^1^Bioactive Resources for Innovative Clinical Applications Research Unit, Chulalongkorn University, Phayathai Road, Pathumwan, Bangkok 10330, Thailand; ^2^Department of Pharmacy Practice, Faculty of Pharmaceutical Sciences, Chulalongkorn University, Phayathai Road, Pathumwan, Bangkok 10330, Thailand; ^3^Department of Chemical Engineering, Faculty of Engineering, Chulalongkorn University, Phayathai Road, Pathumwan, Bangkok 10330, Thailand; ^4^Department of Pharmaceutical Technology and Drug Delivery System Excellence Center, Faculty of Pharmaceutical Sciences, Prince of Songkla University, Hat Yai, Songkhla 90110, Thailand

## Abstract

The genipin-cross-linked silk sericin/poly(vinyl alcohol) (PVA) films were developed aiming to be applied as two-dimensional wound dressings for the treatment of superficial wounds. The effects of genipin cross-linking concentration on the physical and biological properties of the films were investigated. The genipin-cross-linked silk sericin/PVA films showed the increased surface density, tensile strength, and percentage of elongation, but decreased percentage of light transmission, water vapor transmission rate, and water swelling, compared to the non-cross-linked films. This explained that the cross-linking bonds between genipin and silk sericin would reduce the mobility of molecular chains within the films, resulting in the more rigid molecular structure. Silk sericin was released from the genipin-cross-linked films in a sustained manner. In addition, either L929 mouse fibroblast or HaCat keratinocyte cells showed high percentage of viability when cultured on the silk sericin/PVA films cross-linked with 0.075 and 0.1% w/v genipin. The *in vivo* safety test performed according to ISO 10993-6 confirmed that the genipin-cross-linked silk sericin/PVA films were safe for the medical usages. The efficacy of the films for the treatment of superficial skin wounds will be further investigated *in vivo* and clinically. The genipin-cross-linked silk sericin/PVA films would be promising choices of two-dimensional wound dressings for the treatment of superficial wounds.

## 1. Introduction

Epidermal damage as a result of ulcer, burn, or other traumatic incidents requires the treatment with wound dressing for the accelerated regeneration with functional satisfaction. Roles of wound dressing are to protect the wound and provide the optimal microenvironment, as well as activate cellular responses [1]. Despite the availability of numerous types of wound dressings, there is still a high demand for the development of more effective and affordable ones. Wound dressings made of natural proteins or polysaccharides (e.g., collagen, calcium alginate, chitin, and chitosan) and synthetic materials (e.g., silicone gel, poly(ethylene glycol), and poly(l-leucine)) have been widely studied in research area and also used clinically [2, 3]. Silk protein sericin, derived from the silkworm cocoon, has been recently investigated for possible new applications in the biomedical field [4–9]. We previously found that silk sericin could activate collagen production in wounds, which subsequently induced epithelialization [4–6]. It was also reported that silk sericin-treated wounds showed much lesser inflammatory reactions than the wounds treated with cream base (formula control) which showed acute inflammation [4]. Furthermore, silk sericin could promote the attachment and proliferation of human skin fibroblasts and keratinocytes [10–14]. These properties contributed to the excellent suitability of silk sericin as a wound dressing material. 

In order to fabricate wound dressing, silk sericin itself forms fragile materials that are not suitable for the medical usages. Our previous study reported that silk sericin could form strong and stable scaffolds by combining it with poly(vinyl alcohol) (PVA), glycerin (as a plasticizer), and genipin (as a cross-linking agent) [15]. Genipin is a natural cross-linking agent which abundantly found in gardenia fruit extract. It can cross-link proteins including silk sericin by a spontaneous reaction [16]. The three-dimensional genipin-cross-linked silk sericin/PVA scaffolds exhibited good physical and mechanical properties and potentially healed the partial- or full-thickness skin wounds [15, 17]. However, for the healing of epidermal or superficial wounds, two-dimensional wound dressings are rather suitable than the three-dimensional ones. As a novelty of this study, the two-dimensional genipin-cross-linked silk sericin/PVA films fabricated were aimed to be applied for the treatment of superficial wounds. The effects of genipin concentration on properties of the films were investigated. Physical and chemical properties of the genipin-cross-linked silk sericin/PVA films including surface density, cross-linking degree, water contact angle, light and water vapor transmission, humidity absorption, water swelling ability, and chemical structure as well as mechanical properties were characterized. The *in vitro* release test of silk sericin from the films and the degradation test of the films were performed. In addition, the viability, nitric oxide, and collagen production of L929 mouse fibroblast and HaCat keratinocyte cells cultured on the films were assessed. Finally, the *in vivo* test on safety of the films was performed according to ISO 10993-6: Biological evaluation of medical devices.

This preliminary study would be beneficial for further *in vivo* and clinical investigations on efficacy of the genipin-cross-linked silk sericin/PVA films as two-dimensional dressings for the treatment of superficial wounds. 

## 2. Materials and Methods

### 2.1. Materials

The fresh bivoltine white-shell cocoons of *Bombyx mori* produced in a controlled environment were kindly supplied by Chul Thai Silk Co., Ltd. (Petchabun province, Thailand). Poly(vinyl alcohol) (PVA, MW 77,000–82,000) was purchased from Ajax Finechem (New South Wales, Australia). Genipin was obtained from Wako Pure Chemical Industries, Ltd., Tokyo, Japan, while glycerin was purchased from Sigma-Aldrich, St. Louis, MO, USA. Other chemicals were analytical grade and used without further purification. 

### 2.2. Preparation of Silk Sericin

The silkworm cocoons were cut into small pieces, and the silk sericin was extracted using a high temperature and pressure degumming technique [18]. Briefly, silkworm cocoons were put into deionized (DI) water and then autoclaved at 120°C for 60 min. After filtration through a filter paper to remove fibroin fibers, silk sericin solution was concentrated until the desired concentration was achieved (approximately 7% w/v, measured by the BCA Protein Assay Reagent, Pierce, Rockford, IL, USA). The molecular weight of silk sericin obtained was ranging from 25 to 150 kDa.

### 2.3. Fabrication of Genipin-Cross-Linked Silk Sericin/PVA Films

PVA was dissolved at 80°C with a constant stirring for 4 h. Genipin was dissolved in ethyl alcohol at a concentration of 20% w/v. Silk sericin solution (3% w/w), PVA solution (2% w/w), and glycerin solution (1% w/w) were mixed. Genipin solution was added into the mixture to the final concentrations of 0.01, 0.025, 0.05, 0.075, and 0.1% w/v and then mixed at room temperature for 30 min. The mixture was cast onto a petri dish and air-dried for 24 h to obtain the genipin-cross-linked silk sericin/PVA films. The film prepared by the same procedures but without genipin addition was used as a control. 

### 2.4. Morphological Observation

Surface morphology of the films was observed on a scanning electron microscope (SEM, JSM 5410LV, JEOL, Tokyo, Japan) at 15 keV after sputter coated with gold. Surface density of the films was determined by dividing the weight with the dimension (*n* = 6).

### 2.5. Determination of Cross-Linking Degree

The amount of free NH_2_ groups in the silk sericin/PVA films after cross-linking with genipin was evaluated by 2,4,6-trinitrobenzenesulfonic acid (TNBS) method [19]. Briefly, a known weight of film was treated with 1 mL of 4% w/v sodium hydrogen carbonate (NaHCO_3_, pH 8.5) and 1 mL of 0.5% w/v TNBS at 40°C for 2 h. Then, 2 mL of 6 N hydrochloric acid (HCl) was added to the reacting solution and further incubated at 60°C for 1.5 h. The absorbance of the solution was spectrophotometrically determined at 415 nm after suitable dilution, and the amount of free NH_2_ groups was determined from a standard curve prepared from different concentrations of *β*-alanine. The amount of free NH_2_ groups remained in the cross-linked films was calculated in relative to the non-cross-linked film as a control (*n* = 3).

### 2.6. Determination of Water Contact Angle

Water contact angle of the genipin-cross-linked silk sericin/PVA films was evaluated using a sessile drop method [20]. Thin films of genipin-cross-linked silk sericin/PVA were prepared on glass slides. Deionized water (DI) was dropped onto the films using a syringe, and the water contact angle was measured at 30 s after dropping using a computerized video water contact angle system (CAM, RE0110, UK). The baseline and the tangent were drawn using software, and the contact angles were measured from three different points on the films (*n* = 3). 

### 2.7. Evaluation of Light Transmission and Water Vapor Transmission

Each film was cut into a rectangle (1 cm × 4.5 cm) and carefully placed on the internal side of a spectroscopy cell. Percentage of light transmission of the films was measured between the wavelength of 400 to 800 nm at 40 nm intervals using a UV/V spectrophotometer (PerkinElmer Ltd., Bundesverband Solarwirtschaft, Germany). 

Water vapor transmission rate (WVTR) was determined according to ASTM E96-80 with a slight modification (ASTM, 1989). A container with DI water was closed firmly with a film. Then, the container was placed in a desiccator with silica gel at 37°C. The film was weighed on an analytical balance model AB204-S (Mettler Toledo Inc., Ohio, USA) at the predetermining times. The WVTR was calculated according to (1):

(1) $$\begin{array}{c} \text{WVTR}=\frac{w\times x}{t\times A}, \end{array}$$

where *w* represents weight of the film, *A* represents the permeation area, and *x*/*t* was calculated by a linear regression from the points of weight gain and time during constant rate period (*n* = 6).

### 2.8. Moisture Absorption and Water Swelling Tests

The moisture absorption capability of the films was evaluated by placing the dried films in the desiccators in which the relative humidity was controlled by salt solution. Potassium chloride was used to obtain a relative humidity of 81.47 ± 1.48% at 25°C [21]. The films were removed from the desiccators at the pre-determining times and carefully weighed. The percentage of weight increase of the films was calculated relatively to their initial weights (*n* = 6). Swelling test was carried out according to the method of Mandal et al. with a slight modification [8]. Briefly, a known weight of dried film was immersed in 10 mL of DI water. After 24 h, the films were carefully taken out and weighed in the swollen state. The equilibrium swelling of the films was calculated according to

(2) Degree  of  water  swelling  (%)  =  wt−w0w0×100,

where *w*_0_ and *w*_*t*_ represent weights of the dried and swollen films, respectively (*n* = 6). 

### 2.9. Mechanical Test

The tensile test was performed on the films at room temperature using a universal testing machine (Hounsfield H10KM, UK) equipped with a 10 kN load cell at a constant rate of 30 mm/min. The curves of force as a function of deformation (mm) were automatically recorded by the software. The tensile strength (N/mm^2^) and percentage of elongation at break were calculated according to the ASTM D638-01 method (*n* = 6). 

### 2.10. FT-IR Measurement

The functional groups presented in the films were examined using fourier transform infrared (FT-IR) spectroscopy (PerkinElmer, USA) of dried samples. The information on structural contribution was collected in the FT-IR analysis using PerkinElmer Spectrum GX (FT-IR system). All spectra were recorded in the wave number range from 4000 to 650 cm^−1^ at the resolution of 4 cm^−1^. The FT-IR analysis was based on the identification of absorption bands concerned with the vibrations of functional groups presented in the samples.

### 2.11. *In Vitro* Release Test of Silk Sericin from Films

The films were placed into phosphate-buffered saline solution (PBS, pH 7.4) at 37°C with a continuous stirring in a closed container. The PBS solutions (1.5 mL) were collected at the predetermined times and the amount of silk sericin released into the solutions was measured using a BCA protein assay kit (Pierce, Rockford, IL, USA). The absorbance of the solution was measured at 562 nm, and the amount of silk sericin was determined from a standard curve prepared from different concentrations of bovine serum albumin (*n* = 3).

### 2.12. *In Vitro* Enzymatic Biodegradation Test

The films were incubated in 1.6 *µ*g/mL lysozyme solution (hen egg white, 92717 Unit/mg, lot no. 1316456, BioChemika, Fluka Sigma-Aldrich, Switzerland) at pH 7.4, 37°C. The enzyme solution was changed every 2 days to ensure continuous enzyme activity. At each interval of time, the remained films were taken out of the solution, rinsed repeatedly with deionized water, and freeze dried. The dried films were weighed, and the percentage of weight loss was calculated as follows:

(3) Percentage  of  weight  loss  (%)  =  (W0−WtW0)×100,

where *W*_0_ and *W*_*t*_ represent the initial weight of the film before degradation and the weight of the film after degradation at different time intervals, respectively (*n* = 3).

### 2.13. Cell Viability Test

The films were sterilized by ethylene oxide gas at 55°C before cell culture. Either L929 mouse fibroblast or HaCat keratinocyte cells were seeded onto the sterilized films at a density of 5 × 10^5^ cells/film and cultured in Dulbecco's Modified Eagle Medium (DMEM) supplemented with 10% v/v fetal bovine serum (FBS) and 100 U/mL penicillin/streptomycin at 37°C, 5% CO_2_. After 24 and 72 h of culture, the number of cells was quantified using the conventional 3-(4,5-dimethylthiazol-2-yl)-2,5-diphenyltetra zolium bromide (MTT) assay (*n* = 3) [22]. Morphology of cells was observed under optical microscope. 

### 2.14. Nitric Oxide Assay

L929 cells were cultured on the films at the same conditions as those of viability test. After 24 and 72 h of culture, nitric oxide (NO) levels were determined by the Griess reaction. In brief, 100 *μ*L Griess reagent (1% v/v N-(1-naphthyl)-ethylenediamine dihydrochloride and 1% v/v sulfanilamide in 2.5% v/v phosphoric acid) was mixed with an equal volume of medium supernatant. The resulting solution yields a pink solution for a positive result and a yellow solution for a negative result. The absorbance of reacting solution was measured at 570 nm based on a standard curve of sodium nitrate (NaNO_3_) (*n* = 3).

### 2.15. Determination of Soluble Collagen Production

L929 cells were cultured on the films at the same conditions as those of viability test. After 24 and 72 h of culture, the medium supernatants were collected. The total amount of soluble type I collagen was assayed using the Sircol collagen assay kit (Biocolor, UK). The absorbance was determined by a microplate reader (Biohit 830, Biohit) at a wavelength of 500 nm. The amount of collagen was calculated based on a standard curve of soluble collagen (type I collagen standard from American disease-free animals) (*n* = 3). 

### 2.16. *In Vivo* Test on Safety of the Films (ISO 10993-6 Standard)

*In vivo* test was approved by the Ethics Committee of the Faculty of Medicine, Chulalongkorn University. The animal experiments were performed according to Chulalongkorn University Animal Care and Use Committee (CU-ACUC) under standard sterile conditions. The implantation of non-cross-linked and 0.1% w/v genipin-cross-linked silk sericin/PVA films (2 × 2 cm^2^) into the subcutaneous tissue of female Wistar rats (8-week-old, 200–300 g) was carried out. Sofra tulle, a clinically available dressing, consists of a cotton leno weave fabric, impregnated with a base composed of white soft paraffin, anhydrous lanolin, and 1.0% w/w framycetin sulphate, was used as a control. Briefly, the rats were anesthetized, shaved the hair, and disinfected with 70 vol% ethyl alcohol. A 1 cm skin incision was made to form pockets in the subcutaneous tissue; then, the sample was inserted into each pocket. The wound was closed with 6–0 prolene suture and disinfected with Betadine (povidone-iodine topical antiseptics) solution. After 3, 7, 14, and 28 days of implantation, the rats were sacrificed with an overdose of thiopental sodium. The samples and surrounding tissue were retrieved, fixed with 10 vol% formalin solution, and embedded in paraffin. The paraffin-embedded samples were sectioned and stained with Hematoxylin and Eosin (H&E). For histological assessment, the H&E slides were semiquantitatively scored following ISO 10993-6. Inflammatory cell types, neovascularization, fibrosis, and fatty infiltrate were scored by one pathologist at two different times. Intensity of inflammatory cells, neovascularization, fibrosis, and fatty infiltrate was recorded using 0–4 scales (0 = not observed, 1 = rare, 2 = minimal, 3 = heavily infiltrate, and 4 = packed infiltrate). The final score was calculated according to (3) and classified as follows: 0.0–2.9 (sample is nonirritant), 3.0–8.9 (sample is slight irritant), 9.0–15 (sample is moderate irritant), and >15 (sample is severely irritant). The level of irritation was compared to that of the control (Sofra tulle),

(4) Final  score=[2It+Nt]−[2Ic+Nc],

where *I*_*i*_ is the total number of polymorphonuclear cell, lymphocytes, plasma cells, macrophages, giant cells, and necrosis of sample *i* (*i* = test sample (*t*) and control (*c*)) and *N*_*i*_ is the total number of neovascularization, fibrosis, and fatty infiltrate of sample *i* (*i* = test sample (*t*) and control (*c*)).

### 2.17. Statistical Analysis

All quantitative data were shown as mean ± SD. For physical characterization, the statistical significance was determined by paired and unpaired Student's *t*-tests along with ANOVA. For *in vivo *study, all treatment groups were compared by ANOVA, and the differences between groups at different time points were compared by post hoc *t*-test. A value of *P* < 0.05 was considered to be significant.

## 3. Results

### 3.1. Surface Morphology and Density of the Films

Surface morphologies, for example, surface roughness, of non-cross-linked and genipin-cross-linked silk sericin/PVA films were similar (unpublished data). Surface density of the films is presented in Table 1. The surface densities of all genipin-cross-linked silk sericin/PVA films (1.33–1.35 mg/mm^3^) were significantly higher than that of the non-cross-linked films (1.28 mg/mm^3^). By comparing the genipin-cross-linked films, their surface densities were not significantly different. 

### 3.2. Cross-Linking Degree of the Films

Cross-linking degree of the films was increased with the increasing concentration of genipin, as shown in Table 1. The films cross-linked with 0.05, 0.075, and 0.1% w/v genipin showed significantly higher cross-linking degree (22–38%), comparing with those cross-linked with lower concentration of genipin (13–18%).

### 3.3. Water Contact Angle of the Films

Water contact angles of the non-cross-linked and genipin-cross-linked silk sericin/PVA films were around 50–55 degrees, as presented in Table 1. The significant difference in water contact angle among the films was not observed. 

### 3.4. Light and Water Vapor Transmission of the Films

**Figure 1:**
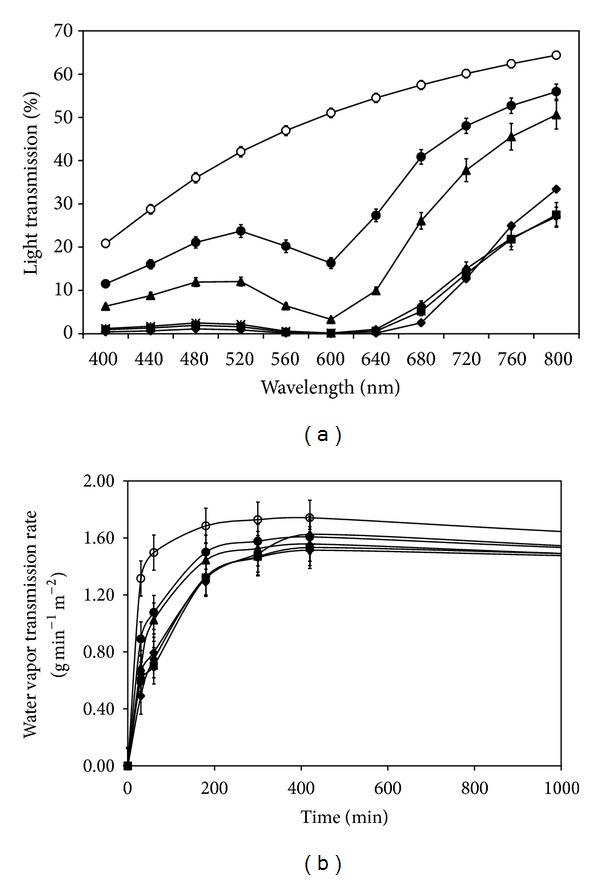
(a) Percentage of light transmission and (b) water vapor transmission rate of silk sericin/PVA films cross-linked with genipin at different concentrations: (○) 0, (•) 0.01, (▲) 0.025, (*◆*) 0.05, (■) 0.075, and (∗) 0.1% w/v. *P < 0.05, significant against the values of non-cross-linked films at corresponding time.

Figure 1(a) shows the percentage of light transmission through the films. For the non-cross-linked films, the percentage of light transmission was gradually increased from 20% up to 60% along the increasing wavelength from 400 to 800 nm. The increasing percentage of light transmission along the increasing wavelength was also observed for the genipin-cross-linked films; however, their light transmission percentages tended to drop at the wavelength around 520–680 nm. Every genipin-cross-linked film showed the minimum percentage of light transmission at 600 nm. The silk sericin/PVA films cross-linked with a higher concentration of genipin showed less percentage of light transmission than those cross-linked with lower concentration of genipin or without cross-linking. 

Figure 1(b) shows the water vapor transmission rate (WVTR) through the films. WVTR through all films was dramatically increased initially and became stable thereafter. All genipin-cross-linked silk sericin/PVA films showed slower WVTR than the non-cross-linked films along the period. The films cross-linked with 0.01 and 0.025% w/v genipin tended to have faster WVTR than those cross-linked with higher concentration of genipin, particularly at the initial period. 

### 3.5. Moisture Absorption and Water Swelling Ability of the Films

**Figure 2:**
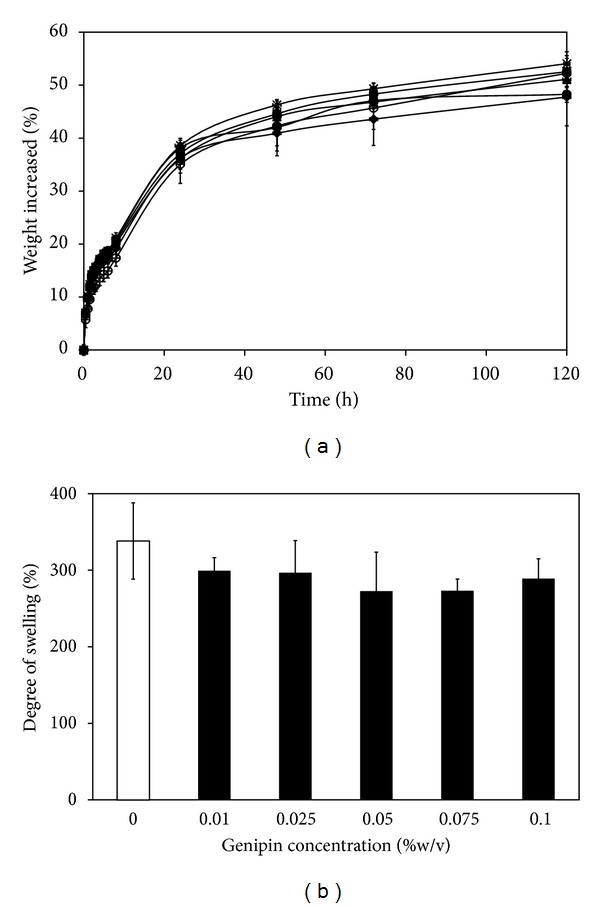
(a) Moisture absorption rate and (b) percentage of water swelling of silk sericin/PVA films cross-linked with genipin at different concentrations: (○) 0, (•) 0.01, (▲) 0.025, (*◆*) 0.05, (■) 0.075, and (∗) 0.1% w/v.

Moisture absorption ability of the films is shown in Figure 2(a). The percentage of weight increased of all films rose from 10% to around 50% along the increasing time. The difference in moisture absorption among the films was not observed.  Figure 2(b) shows the percentage of water swelling of the films. All genipin-cross-linked silk sericin/PVA films showed slightly lower percentage of water swelling, comparing with the non-cross-linked films, but the significant difference was not observed. 

### 3.6. Tensile Strength and Percentage of Elongation of the Films

Table 2 presents the tensile strength and percentage of elongation of the films and Sofra tulle. Increasing concentration of genipin resulted in the higher tensile strength and percentage of elongation of the films. The silk sericin/PVA films cross-linked with 0.05, 0.075, and 0.1% w/v genipin showed significantly higher tensile strength and percentage of elongation than those cross-linked with 0.01 and 0.025% w/v genipin or without cross-linking (*P* < 0.05). The cross-linking with 0.01 and 0.025% w/v genipin seemed not to improve both tensile strength and elongation of the films, comparing to the non-cross-linked films. On the other hand, the Sofra tulle, a cotton leno-weave fabric, showed a significantly higher tensile strength (~26.41 N/mm^2^) but lower elongation percentage (~11%) than the films.

### 3.7. FT-IR Spectra of Films

**Figure 3:**
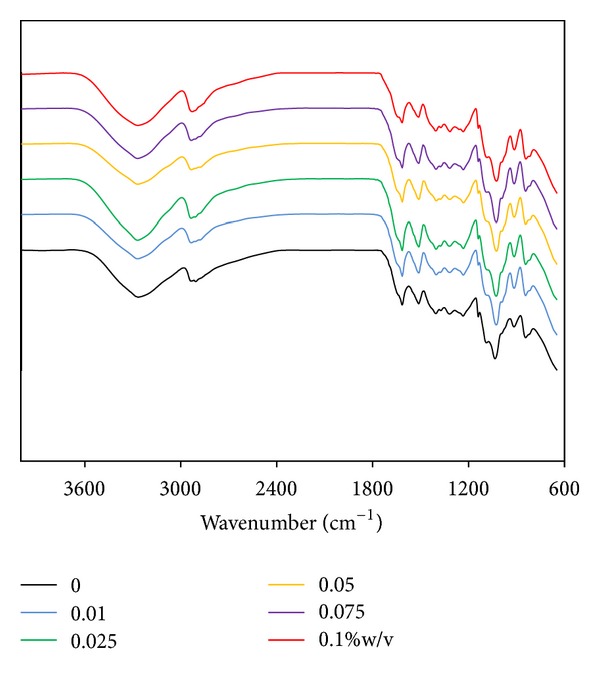
FT-IR spectra of silk sericin/PVA films cross-linked with genipin at different concentrations: (black line) 0, (blue line) 0.01, (green line) 0.025, (yellow line) 0.05, (purple line) 0.075, and (red line) 0.1% w/v.

Figure 3 shows FT-IR spectra of the films. The spectra of all films showed the obvious presence of amide bands of silk sericin protein [23]. The peak positions at 1600 cm^−1^ indicated random coil structure of amide I (C = O stretching). The peaks at around 1530 cm^−1^ were amide II (N–H deformation and C–N stretching), and those at 1250 cm^−1^ indicated random coil structure of amide III (C–N stretching and N–H deformation). The peak positions at 800 cm^−1^ indicated amide V (out-of-plane NH bending). The characteristic absorption peaks of PVA at about 3000 cm^−1^ (–OH stretching) and at about 1082 and 1400 cm^−1^ (–C–O) were observed [24]. The peak positions at around 1150 cm^−1^ (C–O stretching) and 1400 cm^−1^ (C–O–H bending) indicated the characteristics of glycerin [25]. It was observed that the height of peaks at 900 and 1000 cm^−1^ of all genpin-cross-linked films was increased, comparing to those of non-cross-linked film. These peaks may indicate the intramolecular and intermolecular cross-linking bonds in which genipin had a heterocyclic structure with primary amine groups [26].

### 3.8. *In Vitro* Release Profiles of Silk Sericin from Films

**Figure 4:**
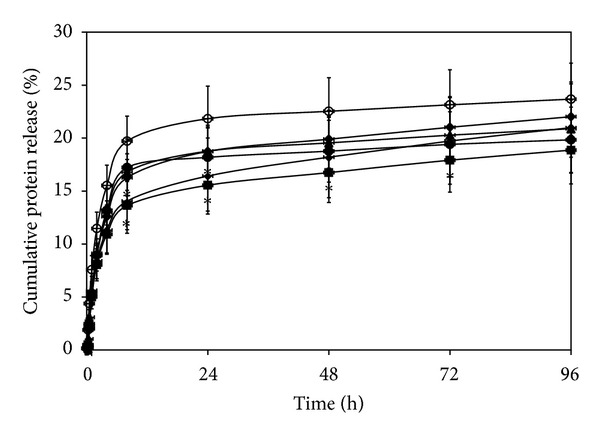
*In vitro* release profiles of silk sericin from the films cross-linked with genipin at different concentrations: (○) 0, (•) 0.01, (▲) 0.025, (*◆*) 0.05, (■) 0.075, and (∗) 0.1% w/v after incubated in a phosphate-buffered saline solution (PBS, pH 7.4) at 37°C and assessed by a BCA protein assay kit. *P < 0.05, significant against the values of non-cross-linked films at corresponding time.

*In vitro* release profiles of silk sericin from the films are shown in Figure 4. At initial time, a burst release of silk sericin was observed for all films, particularly for the films without cross-linking or those cross-linked with lower concentration of genipin (0.01, 0.025, and 0.05% w/v). However, the amount of silk sericin released was reduced and became stable thereafter. The films cross-linked with 0.075 and 0.1% w/v genipin showed less amount of silk sericin release than those cross-linked with lower concentration of genipin. 

### 3.9. *In Vitro* Degradation of the Films

**Figure 5:**
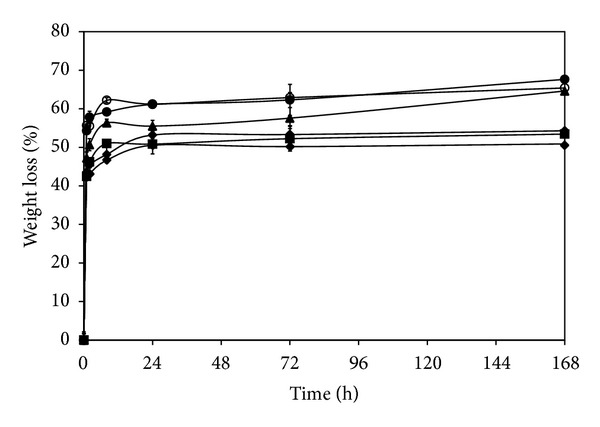
*In vitro* degradation profiles of silk sericin/PVA films cross-linked with genipin at different concentrations: (○) 0, (•) 0.01, (▲) 0.025, (*◆*) 0.05, (■) 0.075, and (∗) 0.1% w/v after incubated in 1.6 *µ*g/mL lysozyme solution (pH 7.4) at 37°C. *P < 0.05, significant against the values of non-cross-linked films at corresponding time.

Figure 5 shows the degradation profiles of the films. Percentage of weight loss of all films gradually increased along the incubation period. The non-cross-linked films and the films cross-linked with 0.01 and 0.025% w/v genipin showed higher percentage of weight loss than the films cross-linked with 0.05, 0.075, and 0.1% w/v genipin. After 168 h of incubation, the weight losses of non-cross-linked films and the films cross-linked with 0.01 and 0.025% w/v genipin were upto 60%, while those of the films cross-linked with 0.05, 0.075, and 0.1% w/v genipin were around 50%.

### 3.10. Viability of Cells Cultured on the Films

**Figure 6:**
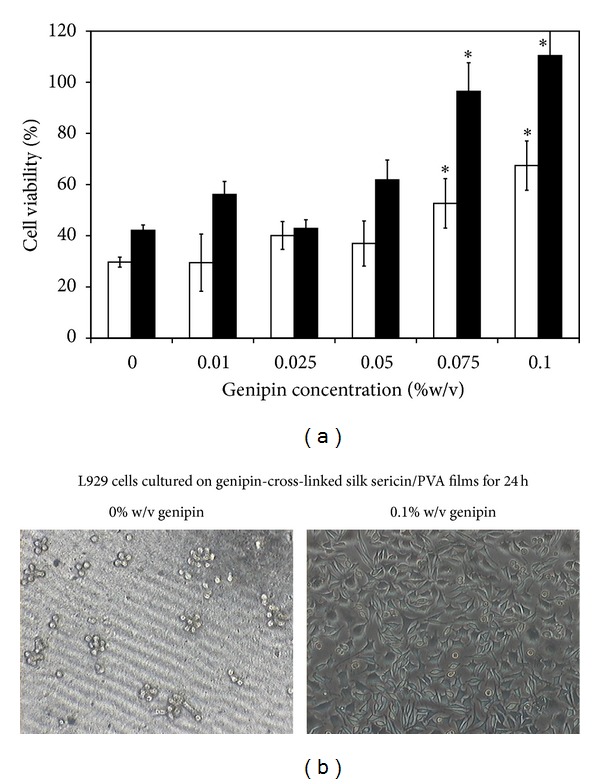
(a) Viability of L929 mouse fibroblast cells cultured on silk sericin/PVA films cross-linked with genipin at different concentrations (0, 0.01, 0.025, 0.05, 0.075, and 0.1% w/v) for (□) 24 h and (■) 72 h. (b) Morphology of L929 cells cultured on non-cross-linked silk sericin/PVA films and silk sericin/PVA films cross-linked with 0.1% w/v genipin for 24 h. *P < 0.05, significant against the values of non-cross-linked films.

**Figure 7:**
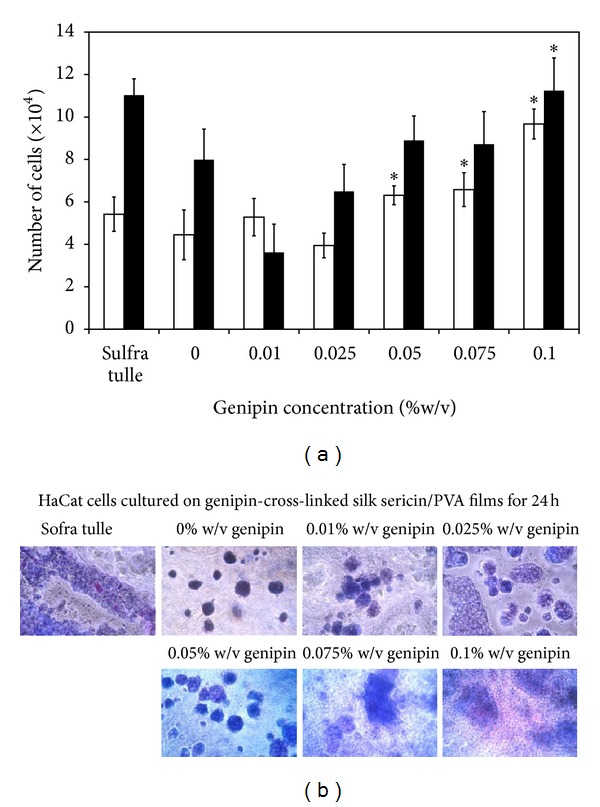
(a) Number of HaCat keratinocyte cells cultured on silk sericin/PVA films cross-linked with genipin at different concentrations (0, 0.01, 0.025, 0.05, 0.075, and 0.1% w/v) for (□) 24 h and (■) 72 h. (b) MTT-stained HaCat keratinocyte cells cultured on the films for 24 h. *P < 0.05, significant against the values of non-cross-linked films.

Figure 6(a) shows the viability of L929 mouse fibroblast cells cultured on the films. After 24 and 72 h of culture, the higher percentage of cell viability was found on the films cross-linked with 0.075 and 0.1% w/v genipin, comparing to those of non-cross-linked film and the films cross-linked with lower concentration of genipin (*P* < 0.05). Morphology of cells was round when cultured on the non-cross-linked films, while those cultured on the films cross-linked with 0.1% w/v genipin showed spindle shape (Figure 6(b)). The numbers of HaCat keratinocyte cells cultured on the films for 24 and 72 h are shown in Figure 7(a). The trend was similar to that of L929 mouse fibroblast cells culture. The HaCat keratinocyte cells cultured on the films cross-linked with 0.075 and 0.1% w/v genipin showed higher number than those cultured on the films cross-linked with 0.01 and 0.025% w/v genipin. When cultured for 72 h, the number of cells cultured on the films cross-linked with 0.1% w/v genipin was comparable to that of Sofra tulle.  Figure 7(b) presents the images of MTT-stained HaCat cells cultured on the films for 24 h. It can be seen that the extent of MTT-stained cells was consistent with the cell number reported in Figure 7(a). 

### 3.11. Production of NO and Soluble Collagen

Production of NO and soluble collagen by cells cultured on the films was shown in Figures 7(a) and 7(b), respectively. After 24 h of culture, cells cultured on the films cross-linked with 0.1% w/v genipin produced the highest concentration of NO, while those cultured on other films seemed to produce very low concentration of NO. After 72 h of culture, the production of NO of cells cultured on the genipin-cross-linked films was significantly higher than that of non-cross-linked film (*P* < 0.05). The production of soluble collagen was seen only for cells cultured on the genipin-cross-linked films and that tended to be higher for the films cross-linked with genipin at higher concentration. The soluble collagen was not detectable for cells cultured on non-cross-linked films at both 24 and 72 h of culture.

### 3.12. *In Vivo* Safety of the Films

**Figure 8:**
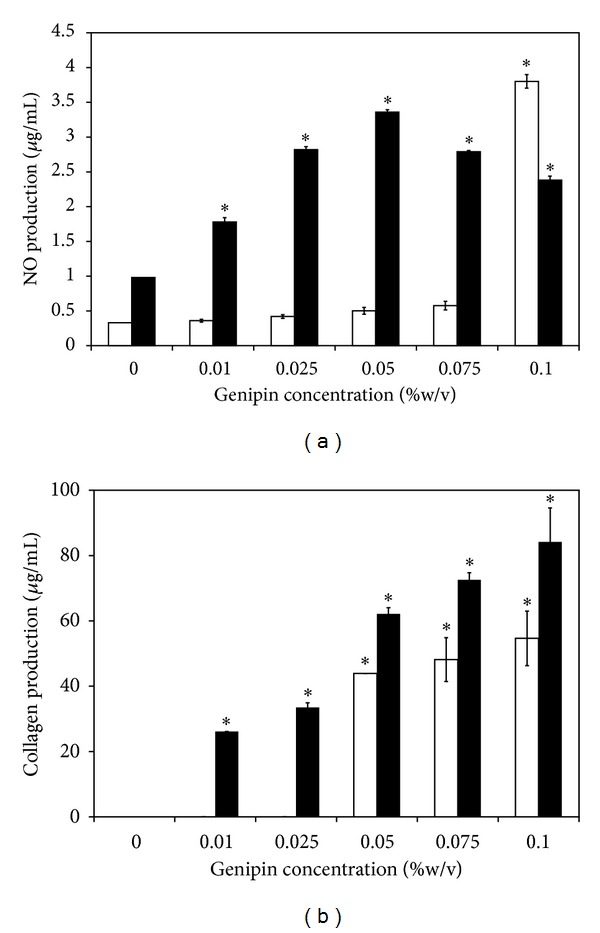
(a) NO and (b) collagen production of L929 cells cultured on silk sericin/PVA films cross-linked with genipin at different concentrations (0, 0.01, 0.025, 0.05, 0.075, and 0.1% w/v) for (□) 24 h and (■) 72 h. *P < 0.05, significant against the values of non-cross-linked films.

**Figure 9:**
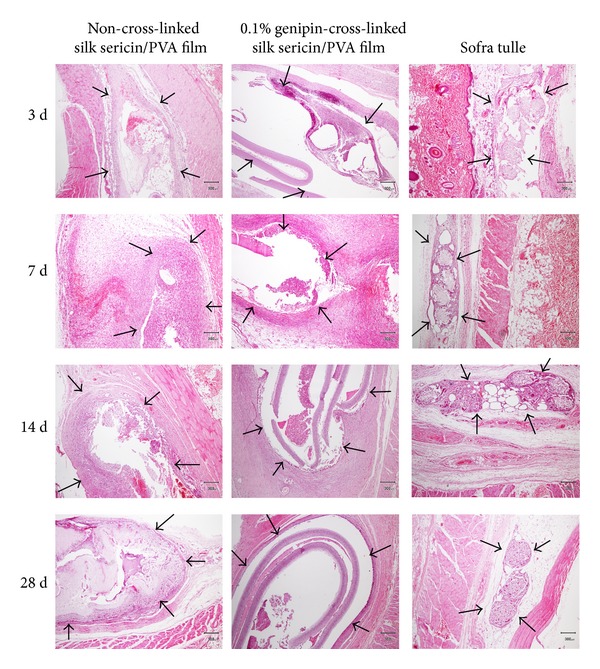
Microscopic images of H&E-stained sections of non-cross-linked silk sericin/PVA films, 0.1% w/v genipin-cross-linked silk sericin/PVA films, and Sofra tulle after subcutaneous implantation for 3, 7, 14, and 28 days. Scale bar = 300 *µ*m, arrow: interface between sample implanted and surrounding tissue.

**Figure 10:**
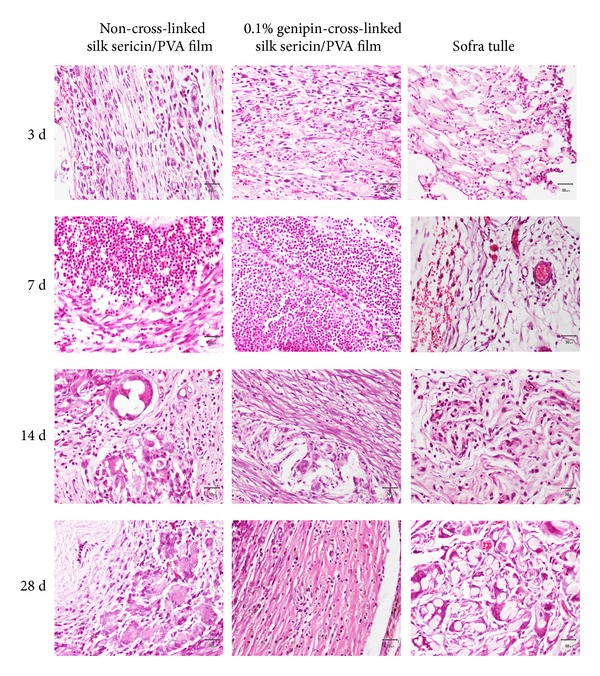
Microscopic images of H&E-stained sections indicating inflammatory cells in non-cross-linked silk sericin/PVA films, 0.1% w/v genipin-cross-linked silk sericin/PVA films, and Sofra tulle after subcutaneous implantation for 3, 7, 14, and 28 days. Scale bar = 30 *µ*m.

The rats which received the implantation of all samples were healthy throughout the implantation period and no signs of inflammation (i.e., redness, swelling, pain, and heat) were observed.  Figure 8 showed the images of H&E-stained sections of non-cross-linked, 0.1% w/v genipin-cross-linked silk sericin/PVA films, and Sofra tulle. The arrows indicated the interface between sample implanted and surrounding tissue. No excessive inflammatory reaction was detected around the implantation sites. The infiltration of inflammatory cells into the samples implanted was shown in Figure 9. The number of inflammatory cells infiltrated into the non-cross-linked and 0.1% w/v genipin-cross-linked silk sericin/PVA films was slightly higher than that of Sofra tulle along the implantation period. The intensity of inflammatory cells, necrosis, fibrosis, neovascularization, and fatty infiltrate was graded as presented in Table 3. After 3 days of implantation, polymorphonuclear cells were packed infiltrated into the non-cross-linked and 0.1% w/v genipin-cross-linked silk sericin/PVA films and heavily infiltrated into the Sofra tulle. However, the intensity of polymorphonuclear cells was gradually reduced thereafter. Lymphocytes, macrophages, and neovascularization were found along the implantation period. Heavy infiltration of giant cells and fibrosis was observed particularly after 14 and 28 days of implantation (see Figure 10). Sofra tulle implantation showed more intensity of fatty infiltrate than both films implantation. On the other hand, plasma cells were not observed for all samples along the implantation period. It was evaluated that the implantation of both films was non- to slightly irritant, relative to the Sofra tulle as a control (Table 4).

## 4. Discussion

Wound dressing made of silk sericin would accelerate wound healing because silk sericin could promote the attachment and proliferation of fibroblasts and keratinocytes [10–14] and induce the collagen production and epithelialization [4–6]. We previously reported that the three-dimensional genipin-cross-linked silk sericin/PVA scaffolds exhibited good physical properties and potentially healed the partial- or full-thickness skin wounds [15, 16]. In this study, the two-dimensional genipin-cross-linked silk sericin/PVA films developed were aimed to be applied for the treatment of superficial wounds. The effects of genipin cross-linking on the physical and biological properties of silk sericin/PVA films were reported. Herein, it was shown that the increasing concentration of genipin resulted in the higher cross-linking degree of the films (Table 1). This was correlated with the increased surface density (Table 1) and mechanical properties (Table 2), but decreased percentage of light transmission and WVTR (Figure 1) and poorer water swelling ability (Figure 2(b)) of the genipin-cross-linked silk sericin/PVA films. 

The cross-linking mechanism of genipin with a methylamine of proteins or other molecules containing primary amines was proposed [26]. The reaction occurred through a nucleophilic attack of the primary amine on the C3 carbon of genipin. This caused an opening of the dihydropyran ring, followed by an attack on the resulting aldehyde group by the secondary amine group. The final step of the formation of cross-linking bonds was the dimerization produced by radical reactions. The intra- and intermolecular cross-linking bonds in which genipin had a heterocyclic structure with the molecules containing primary amine groups were formed. The increased height of peaks of FT-IR spectra at 900 and 1000 cm^−1^ may confirm these intra- and inter-molecular cross-linking bonds of the genipin-cross-linked silk sericin/PVA films (Figure 3). 

The increased density and improved mechanical properties of the films as a result of cross-linking were reported elsewhere [27, 28]. Huang et al. have studied the effects of calcium chloride cross-linking on structure and properties of waterborne polyurethane-carboxymethylated guar gum films [28]. The cross-linked films formed a relatively dense architecture, which led to better miscibility and higher tensile strength. On the other hand, the genipin-cross-linked films showed lower percentage of light and water vapor transmission because the cross-linking bonds reduced the mobility of molecular chains within the films [29]. The rigid structure of the cross-linked films would obstruct the transmission of light and water vapor. This behavior has also been reported in alginate films with higher concentrations of calcium [30]. We here also elucidated the effect of genipin cross-linking on the degradation behavior of the silk sericin/PVA films. It was found that the genipin-cross-linking, particularly at high concentration, could prolong the degradation of the silk sericin/PVA films (Figure 5).

The films which are capable of swelling would support the adsorption of proteins or bioactive molecules from culture medium or body fluid and subsequently promote bioactivity of cells [31]. In this study, the genipin-cross-linking slightly reduced the water swelling ability of the films. It is possible that the rigid molecular chains of the cross-linked films impeded the penetration of water molecules through the films. The effects of chemical cross-linking on the molecular crystallinity and diffusion coefficient of water were also reported by Hasimi et al. [32]. However, from our results, the significant difference in water swelling ability between the genipin-cross-linked and non-cross-linked films was not observed. Thus, the genipin-cross-linking would not diminish the capability of the silk sericin/PVA films to adsorb proteins for biological signaling. 

The release of silk sericin from the films was possibly governed by two main mechanisms, which are diffusion and material degradation [33]. The former mechanism was seen from the burst release of non-cross-linked silk sericin, while the latter mechanism explained the release of the genipin-cross-linked silk sericin. Initially, the small amount of silk sericin was diffusionally released from the films cross-linked with 0.075 and 0.1% genipin (Figure 4) because most of the silk sericin was cross-linked with genipin. On the other hand, high amount of non-cross-linked silk sericin found in the films cross-linked with lower concentration of genipin (0.01–0.05%) was diffusionally released at initiation. However, thereafter, the genipin-cross-linked silk sericin was sustained and released from all films along the degrading of the films. 

For the biological activities, it was found that L929 mouse fibroblast cells cultured on the silk sericin/PVA films cross-linked with high concentration of genipin (0.075 and 0.1% w/v) showed higher percentage of viability (Figure 6(a)) and produced higher concentrations of NO and soluble collagen (Figure 8). Nitric oxide is usually produced during inflammatory conditions by the inducible isoform of the enzyme NO synthase [34]. It was reported that some concentrations of NO can be potentially beneficial for cell activities [35–38]. Witte et al. reported that the synthesis of fibroblastic collagen together with total protein was enhanced in the presence of low concentrations of NO [34, 36]. However, it should be noted that there are adverse effects of NO on wound healing. The excessive generation of intracellular ROS and NOS in the skin may result in subsequent oxidative stress damage and apoptosis. NO overexpression may be involved directly or indirectly, through production of peroxynitrite, in the pathogenesis and delayed healing due to the impaired vasculature, inflammation, and toxicity. In addition to the L929 mouse fibroblast cells, the viability of HaCat keratinocyte cells when cultured on the films was shown (Figure 7). The results further confirmed that the films cross-linked with high concentration of genipin promoted cell proliferation. It might be that the films cross-linked with 0.075 and 0.1% w/v genipin could entrap high amount of silk sericin and sustained its release which probably improve biological properties of the films. It has been reported that silk sericin could activate collagen production [4–6] and promote attachment and proliferation of human skin fibroblasts and keratinocytes [10–14].

The *in vivo* evaluation on safety of the films was performed according to ISO 10993-6, comparing with Sofra tulle, to confirm their suitability for medical applications. It was shown that the implantation of the 0.1% w/v genipin-cross-linked silk sericin/PVA films was evaluated as slightly irritant which is clinically acceptable for topical applications, relative to the Sofra tulle implantation (Table 4). Therefore, the 0.1% w/v genipin-cross-linked silk sericin/PVA films would be safe for the further *in vivo* and clinical efficacy tests as two-dimensional dressings for the treatment of superficial wounds.

It was concluded from this study that the silk sericin/PVA films cross-linked with genipin, particularly at high concentration, showed good physical and biological properties as well as the sustained release of sericin which would be advantageous for the healing of superficial wounds.

## 5. Conclusion

The silk sericin/PVA films cross-linked with genipin showed increased surface density and mechanical properties, but decreased percentage of light transmission and WVTR and poorer water swelling ability, comparing with the non-cross-linked films. Silk sericin was released from the genipin-cross-linked films in a sustained manner. Either L929 mouse fibroblast or HaCat keratinocyte cells cultured on the silk sericin/PVA films cross-linked with high concentration of genipin (0.075 and 0.1% w/v) showed higher percentage of viability and produced higher concentrations of NO and soluble collagen. The *in vivo* safety test performed according to ISO 10993-6 confirmed that the 0.1% w/v genipin-cross-linked silk sericin/PVA films would be safe for the medical usages. Therefore, the 0.1% w/v genipin-cross-linked silk sericin/PVA films would be promising choices of two-dimensional wound dressings for the treatment of superficial wounds. 

## Figures and Tables

**Table 1:** Surface density, degree of cross-link, and water contact angle of silk sericin/PVA films cross-linked with genipin at different concentrations (0, 0.01, 0.025, 0.05, 0.075, and 0.1% w/v).

Genipin concentration (% w/v)	Surface density (mg/mm^3^)	Degree of cross-link (%)	Contact angle (°)
0	1.28 ± 0.04	—	54.91 ± 4.29
0.010	1.33 ± 0.06*	13.84 ± 2.59	54.71 ± 9.74
0.025	1.33 ± 0.03*	18.82 ± 4.00	52.06 ± 7.66
0.050	1.36 ± 0.09*	22.64 ± 1.89^†^	53.85 ± 3.29
0.075	1.35 ± 0.07*	29.75 ± 6.46^†^	52.63 ± 3.57
0.100	1.35 ± 0.10*	38.97 ± 4.51^†^	54.04 ± 10.10

*P < 0.05, significant against the value of non-cross-linked film.

^†^ *P* < 0.05, significant against the value of films cross-linked with 0.01% w/v.

**Table 2:** Tensile strength and percentage of elongation of silk sericin/PVA films cross-linked with genipin at different concentrations (0, 0.01, 0.025, 0.05, 0.075, and 0.1% w/v).

Genipin concentration (% w/v)	Tensile strength (N/mm^2^)	Elongation (%)
Sofra tulle	26.41 ± 2.59	11.07 ± 1.71
0	4.97 ± 0.59	188.54 ± 55.22
0.010	4.66 ± 0.72	156.50 ± 31.93
0.025	5.82 ± 0.91	198.68 ± 55.64
0.50	7.14 ± 0.57*	309.60 ± 63.36*
0.075	8.17 ± 2.15*	331.98 ± 55.02*
0.100	9.17 ± 1.37*	361.98 ± 53.85*

*P < 0.05, significant against the values of non-cross-linked film.

**Table 3:** Average intensity of inflammatory cells, necrosis, fibrosis, neovascularization, and fatty infiltrate in non-cross-linked silk sericin/PVA films, 0.1% w/v genipin-cross-linked silk sericin/PVA films, and Sofra tulle after subcutaneous implantation for 3, 7, 14, and 28 days.

	3 d			7 d			14 d			28 d		
	A*	B**	C***	A	B	C	A	B	C	A	B	C
PMN	4^†^	4	3	2.8	3.3	0.8	0.8	3	0	0	3.8	0
Lymphocytes	2.8	2.8	1.5	3	3	2	3.5	3	2.3	3	3	1
Plasma cells	0	0	0	0	0	0	0	0	0	0	0.8	0
Macrophages	2.3	2.8	3	3	3	2	3.3	3	3	3	3	2.3
Giant cells	0	0	0	0	0	2.3	3.3	1.3	3.8	2.3	1	4
Necrosis	1	0	0	1	0	0	1.5	1.5	0	1.5	0	0
Fibrosis	1.8	0.8	1.8	3.3	4	2.8	3.5	4	3	3	3.5	2.5
Neovascularization	3	3	3	3	2.8	2.3	3	3	2.8	1	3	1.3
Fatty infiltrate	0	0	0	0	1	2.3	0	0	2.3	0.5	0	3

*A: non-cross-linked silk sericin/PVA films.

**B: 0.1% w/v genipin-cross-linked silk sericin/PVA films.

***C: Sofra tulle (control).

^†^Intensity 0–4: 0 = not observed, 1 = rare, 2 = minimal, 3 = heavily infiltrate, and 4 = packed infiltrate.

**Table 4:** Level of tissue irritation after subcutaneous implantation with non-cross-linked silk sericin/PVA films and 0.1% w/v genipin-cross-linked silk sericin/PVA films for 3, 7, 14, and 28 days, relative to Sofra tulle as a control.

	Level of irritation	
	Non-cross-linked silk sericin/PVA films	0.1% w/v genipin-cross-linked silk sericin/PVA films
3 d	Slightly irritant	Slightly irritant
7 d	Slightly irritant	Slightly irritant
14 d	Slightly irritant	Slightly irritant
28 d	Nonirritant	Slightly irritant
